# Brief review on the roles of neutrophils in cancer development

**DOI:** 10.1002/JLB.4MR0820-011R

**Published:** 2020-09-24

**Authors:** Wang Long, Jingjing Chen, Chen Gao, Zhi Lin, Xubiao Xie, Helong Dai

**Affiliations:** ^1^ Department of Kidney Transplantation The Second Xiangya Hospital of Central South University Changsha China; ^2^ Department of Pathological Cell Biology Graduate School of Medical and Dental Science Tokyo Medical and Dental University Tokyo Japan; ^3^ Clinical Research Center for Organ Transplantation in Hunan Province Changsha China; ^4^ Clinical Immunology Center Central South University Changsha China

**Keywords:** neutrophils, cancer, MDSC, heterogeneity, neutrophil‐to‐lymphocyte ratio

## Abstract

Neutrophils, which are traditionally regarded as a hallmark of inflammation, are also a member of the intratumoral immune cells. The roles of neutrophils in cancer development are diverse and undefined. So far, they are known to be involved in tumor initiation and tumor cell proliferation and metastasis. They show heterogeneity in both phenotypes and functions during early versus late stage of cancer development. Because they are also associated with the clinical outcomes of various types of solid tumors, cancer treatments that target neutrophils might be highly effective. In this review, we briefly cover the latest findings on the multiple roles of neutrophils in cancer development and point out the future directions as well.

## Graphical Abstract

Reviews how neutrophils support tumor growth, enhance tumor cell proliferation, escort circulating tumor cells for metastasis.

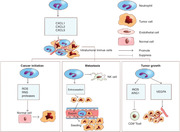

**Abbreviations**
anti‐Gr1: anti‐granulocyte receptor 1
APC: antigen‐presenting cell
ARG1: arginase 1
Bv: Bevacizumab
CCL2: chemokine (C‐C motif) ligand 2
CRP: C‐reactive protein
CTCs: circulating tumor cells
CTLA‐4: cytotoxic T lymphocyte‐associated‐antigen 4
FATP2: fatty acid transporter protein 2
GBM: glioblastoma
G‐CSF: granulocyte‐colony stimulating factor
GM‐CSF: granulocyte‐macrophage‐colony stimulating factor
GMP: granulocyte‐monocyte progenitors
GPI: glycosylphosphatidylinositol
IFN: Interferon
IL‐6: Interleukin‐6
iNOS: inducible nitric oxide synthase
MDSCs: Myeloid‐derived suppressor cells
M‐MDSCs: monocytic myeloid‐derived suppressor cells
MMP‐9: matrix metallopeptidase 9
MPO: myeloperoxidase
NETs: neutrophils extracellular traps
NK: natural killer
NLR: neutrophil‐to‐lymphocyte ratio
PD‐1: programmed cell death protein‐1
PDL1: programmed death ligand 1
PMN‐MDSCs: polymorphonuclear myeloid‐derived suppressor cells
PTEN: phosphatase and tensin homologue
RNS: reactive nitrogen species
ROS: reactive oxygen species
STAT5: signal transducer and activator of transcription 5
TANs: tumor‐associated neutrophils
TGF‐β: transforming growth factor β
Treg: regulatory T cells
VCAM1: vascular cell adhesion molecule 1
VEGFA: vascular endothelial growth factor A
WBCs: white blood cells

## 1 INTRODUCTION

Neutrophils are the most abundant type of WBCs in the human body. The name “neutrophil” is derived from the neutral pink color that these cells develop after histologic staining with H&E. By contrast, basophils are stained a dark blue color and eosinophils are stained bright red. Neutrophils are involved mainly in the innate immune response, during which they develop rapidly but have a short lifespan (8–10 h in mice, 5.4 days in human),^1^, ^2^ which may contribute to the underestimation of the roles they play in cancer development. Bone marrow contributes the most to a steady state of neutrophils formation, but when a large quantity of neutrophils has to be generated because they are being used up during an infection or in cancer, emergency granulopoiesis will take over.^3^ Most of the neutrophils are formed in bone marrow, and only 1–2% will circulate in the peripheral blood.^4^ However, the spleen can also be a potential source for neutrophil formation in some types of cancer.^5^ Neutrophil generation and differentiation rely mainly on G‐CSF,^6^, ^7^, ^8^ whereas other factors like GM‐CSF, IL‐6, and stem cell factor have less effects on neutrophil maturation.^9^, ^10^, ^11^ Neutrophils generation is commonly regarded that they start from granulocyte‐monocyte progenitors (GMP), then differentiate into proliferative neutrophils including myeloblast, promyelocyte, myelocyte, and nonproliferative neutrophils including metamyelocyte banded neutrophils, mature neutrophils, and become circulating neutrophils at last.^12^ Neutrophils are commonly defined with surface markers expression, like GPI‐linked receptor Ly6G in mice or CD66b in human.^13^ Development of single‐cell sequencing in neutrophils deeply elucidated different neutrophils precursors into proneutrophils, immature neutrophils, and mature neutrophils. In bone marrow, mouse proneutrophils are Lin^−^CD117^+^Siglec‐F^−^GR11^+^CD11b^+^CXCR4^+^, human proneutrophils are Lin^−^CD66b^+^CD15^+^CD33^int^CD49d^int^CD101^−^, mouse immature neutrophils are Lin^−^CD115^−^Siglec‐F^−^GR1^+^CD11b^+^Ly6G^+^CXCR2^−^CD101^−^, human immature neutrophils are Lin^−^CD66b^+^CD15^+^CD33^int^CD49d^−^CD101^int^CD10^−^CD16^int^, mouse mature neutrophils are Lin^−^CD115^−^Siglec‐F^−^GR1^+^CD11b^+^Ly6G^+^CXCR2^+^CD101^+^, and human mature neutrophils are Lin^−^CD66b^+^CD15^+^CD33^int^CD49d^−^CD101^int^CD10^+^CD16^hi^.^14^, ^15^ The stimulating factors and cytokines related to neutrophil release from bone marrow into circulating blood include IL‐23, G‐CSF, IL‐17, and CXC chemokine receptors.^16^, ^17^

## 2 NEUTROPHILS FACILITATE CANCER DEVELOPMENT

**FIGURE 1:**
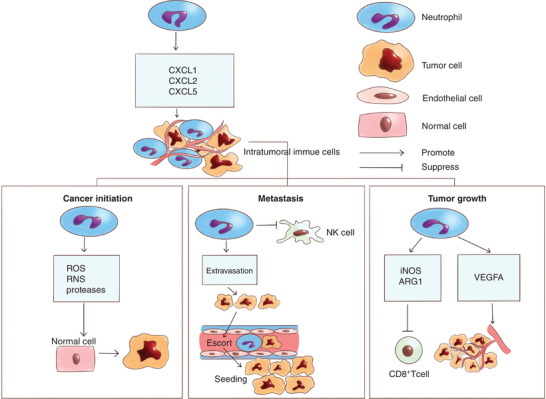
**Neutrophils are attracted by CXCR2 ligands and infiltrate the tumor microenvironment to become intratumoral immune cells**. Neutrophils support cancer development mainly through 3 pathways: (1) their support of cancer initiation through the secretion of reactive oxygen species, reactive nitrogen species, and some proteases; (2) their facilitation of metastasis through the suppression of natural killer cells, enhancement of extravasation, and escorting of tumor cells to spread and seed; and (3) their support of tumor growth through the inhibition of CD8^+^ T cells and promotion of angiogenesis. VEGFA, vascular endothelial growth factor A

Neutrophils are abundant in microenvironment of most of solid tumors.^18^, ^19^, ^20^, ^21^ A bunch of papers have already elucidated that neutrophils or tumor‐associated neutrophils (TANs) in cancer microenvironment or circulating neutrophils are associated with poor patient survival and resistance to cancer treatment including chemotherapy, radiation therapy, and antiangiogenesis therapy.^22^, ^23^, ^24^, ^25^, ^26^ Neutrophils have many roles in cancer development; for example, they may support cancer initiation, promote tumor growth, and contribute to cancer metastasis.^27^, ^28^ Neutrophils support cancer initiation. As one of the inflammatory immune cells, neutrophils express chemokine receptors CXCR1 and CXCR2,^29^ which are attracted by CXCR2 ligands, so that they can infiltrate the tumor microenvironment.^28^, ^30^ Cancer cells express plenty of chemokines like CXCL5, CXCL6, and CXCL8 that recruit neutrophils.^31^ Some of the tumor cells like Lewis lung carcinoma cells express Liver X receptor ligand that recruits neutrophils via CXCR2.^32^ Neutrophils recruited into tumor microenvironment support cancer initiation through the release of reactive oxygen species (ROS), reactive nitrogen species (RNS), or proteases.^33^ Neutrophils facilitate tumor growth. Neutrophils support tumor growth through the promotion of angiogenesis, since neutrophil depletion suppresses vessel formation.^33^ Another critical mechanism is associated with neutrophils extracellular traps (NETs) (been marked like circulating DNA). NETs were found to be a protective component against pathogens, since they are important for neutrophils to isolate cancer cells with antimicrobial factors.^34^, ^35^ Neutrophils also promote tumor cell proliferation through the secretion of inducible NOS (iNOS) and arginase 1 (ARG1) to inhibit CD8^+^ T cells^36^, ^37^ (Fig. 1).

Furthermore, neutrophils promote cancer metastasis through many mechanisms. For example, in the initial stage of metastasis, neutrophils suppress the function of natural killer cells and enhance the extravasation of tumor cells.^38^ At a later disease stage, they interact directly with circulating tumor cells (CTCs) to promote cell cycle progression of those cells and accelerate their seeding.^39^ One paper selected CTCs and CTCs‐associated WBCs in patients with breast cancer and mouse models, with single‐cells RNA sequencing, in which they found that in most of cases, CTCs are associated with neutrophils, and CTCs interaction with neutrophils is mediated by VCAM1.^39^ NETs also can induce cancer cells migration in vitro,^40^ especially large amount of NETs production under infection.^40^, ^41^ Another piece of evidence to support this role of neutrophils is the finding that their depletion through the use of antigranulocyte receptor‐1 (anti‐Gr1) antibodies suppressed metastasis.^42^, ^43^ Surprisingly, studies have also found that the depletion of antimetastatic neutrophils regulated by chemokine (C‐C motif) ligand 2 (CCL2) enhances metastasis.^44^, ^45^ Neutrophils can also promote metastasis through direct interaction with cancer cells.^42^

## 3 HETEROGENEITY OF NEUTROPHILS IN CANCER

It is already broadly agreed that neutrophils play a critical role in cancer development, while antitumoral role of neutrophils is not well understood. The heterogeneity of neutrophils has been reviewed elsewhere.^46^, ^47^, ^48^ Myeloid‐derived suppressor cells (MDSCs), which are a group of myeloid cells that expand under pathologic conditions, such as inflammation, infection, and cancer, express CD11b and Gr1 in mice.^49^, ^50^ There are 2 subtypes of MDSCs based on their level of Gr1 expression; namely, polymorphonuclear myeloid‐derived suppressor cells (PMN‐MDSCs) with CD11b^+^Gr1^high^ expression, and monocytic myeloid‐derived suppressor cells (M‐MDSCs) with CD11b^+^Gr1^low^ expression. Since PMN‐MDSCs share the same marker with neutrophils, they are commonly regarded as pathologic neutrophils. Other than as neutrophils that support tumor growth, PMN‐MDSCs are regarded as immunosuppressive cells.^51^ The immunosuppressive function of PMN‐MDSCs is mediated by many molecules, including iNOS, ARG1, and fatty acid transporter protein 2 (FATP2).^52^, ^53^, ^54^ The regulation of FATP2 in the immunosuppressive function of PMN‐MDSCs was reported recently.^54^ In this study, FATP2 was overexpressed on PMN‐MDSCs only, where it was regulated by GM‐CSF via the activation of STAT5. The accumulation of arachidonic acid and subsequent synthesis of prostaglandin 2 were also involved in the FATP2 regulatory process. Impressively, mice treated with a FATP2 inhibitor together with immune checkpoint blockades exhibited significantly suppressed tumor growth.^54^ Neutrophil also induces antitumor resistance through several pathways. Interaction between neutrophils and cells in tumor microenvironment is involved in antitumor resistance induction.^36^, ^55^, ^56^ A recent study showed that neutrophils also induce antitumor resistance by promoting unconventional CD4^−^CD8^−^ αβT cells polarization in an IFN‐γ‐dependent manner, with the cooperation of macrophages.^57^ Also, neutrophil induced antitumor resistance is related to immune checkpoints blockade^58^, ^59^, ^60^, ^61^ and NOTCH1 signaling activation.^62^, ^63^ Neutrophils drive the accumulation of MDSCs, which leads to antitumor resistance as well.^64^

In cancer development, sometimes immune cells showed different or opposite function at different stages,^65^ like macrophages are antitumoral cells at early stage while tumor supporting function at later stage.^66^ First, in the early stage of human lung cancer, TANs are immunosuppressive instead of stimulative to T cell response, since they can enhance T cell proliferation and IFN‐γ release through the secretion of proinflammatory factors and upexpression of costimulatory molecules on their surface.^67^ These special TANs in the early stage of human lung cancer exhibit a dual characteristics of both neutrophils and APC. They are named APC‐like “hybrid neutrophils,” which can migrate into tumor‐draining lymph nodes and cross‐present antigens to suppress T cells function.^68^ Second, neutrophils may directly kill tumor cells by releasing ROS and RNS.^44^, ^56^, ^69^ In addition, it is published many years ago that TGF‐β blockade is related to neutrophils antitumoral function.^36^ Following TGF‐β blockade, tumor‐associated CD11b^+^Ly6G^+^neutrophils increased rather than Ly6G^−^ macrophages, and these intratumoral neutrophils play a significant role in tumor cytotoxicity of myeloid cells in anti‐TGF‐β treatment in a CD8^+^‐dependent manner.^36^ Three different neutrophils are reported in circulating neutrophils sorted by density properties. Most of circulating neutrophils are high‐density neutrophils in healthy mice, whereas in cancer progressing mice, the majority of circulating neutrophils are tend to be low‐density neutrophils, including immature MDSCs and mature neutrophils derived from high‐density neutrophils in a TGF‐β‐dependent manner.^70^ Except for TGF‐β, IFN signaling is also involved in antitumoral function.^71^, ^72^ Antitumoral neutrophils are able to expand under type I IFN both in mice and human. In the absence of IFN‐β, NET expression reduced with lower tumor cytotoxicity, and reduced IFN‐α and ICAM‐1 expression, resulting in predominant protumor neutrophils.^71^

## 4 NEUTROPHILS INTERACT WITH TUMOR MICROENVIROMENT

Tumor development facilitates neutrophils expansion and function. For example, tumor initiation induces neutrophil emergency granulopoiesis, which enhances the development of neutrophils and suppresses neutrophils retention. Tumor enhances the development of neutrophils by increasing G‐SCF and GM‐SCF level to gravely expand GMP and neutrophil progenitors.^52^, ^73^, ^74^, ^75^, ^76^, ^77^ Many papers elucidated other pathways like expression of KIT (CD117) ligand and CXCR2 receptors by cancer cells enhance response to hypoxia, which disturb neutrophils retention in bone marrow.^78^, ^79^ KRAS signaling in cancer cells and PTEN or SMAD4 depletion will promote GM‐CSF and CXCR2 ligand expression.^80^, ^81^, ^82^

Cancer cells also regulate neutrophils metabolism. Cancer cell can induce NETs production from neutrophils to enhance metabolism, through extracellular RNAs from cancer cells.^83^ Tumor microenvironment also induces metabolically adaptation for neutrophils in mitochondrial metabolism and oxidative phosphorylation to maintain immune suppression.^84^ Different metabolic profiles are associated with the distinct functions of neutrophils. The immature low‐density neutrophils can perform enhanced liver metastasis functions under metabolically challenges such as glucose‐deprived condition, which is owed to their enhanced global bioenergetic capacity.^85^

Besides direct interaction with tumor cells to promote or inhibit tumor development as described above, neutrophils also interact with other cells in tumor microenvironment. Immune checkpoints including cytotoxic T lymphocyte‐associated‐antigen 4 (CTLA‐4), programmed cell death protein‐1 (PD‐1), and programmed death ligand‐1 (PDL1) are critical mechanism for tumor cells to escape from immune system, therefore immune checkpoints blockade is used for the treatment of cancer in clinic. It is reported that immune checkpoint blockade may also inhibit cancer‐associated fibroblast‐induced PDL1 expression on neutrophils, which inhibits T cells activation in hepatocellular carcinomas.^86^ Except for immune checkpoints, regulatory T cells (Treg) also regulate T cells activation and play a crucial role in immunologic escape mediated by multiple molecules including CTLA4, IL‐10, LAG3, CD73, and CD39, whereas CCL2 and CCL17 secreted by neutrophils recruit anti‐inflammatory macrophages and Treg to facilitate tumor growth.^87^, ^88^ PMN‐MDSCs and other protumor neutrophils can induce apoptosis of CD8+ T cells function in a TNF‐α and NO‐dependent manner.^89^ T cells also regulate neutrophils expansion. The absence of γδT cells and neutralizing IL‐17 inhibits the accumulation of neutrophils, indicating that IL‐17 producing γδT cells regulate neutrophils expansion.^52^ Protumor neutrophils can also impair NK cells function via myeloperoxidase (MPO) and hydrogen peroxide secretion and CXCR4 expression.^90^, ^91^, ^92^

## 5 CLINICAL EVALUATION BY NEUTROPHILS

Many studies have found that the neutrophil levels in patients correspond to their clinical outcomes, and the neutrophil‐to‐lymphocyte ratio (NLR) has become the most significant prognostic marker for many types of cancer. An elevated NLR is correlated with worse outcomes in many types of cancer and even reduces the patient's response to some antitumor treatments,^93^, ^94^, ^95^, ^96^, ^97^ indicating that neutrophils may promote, rather than suppress, tumor growth. For example, in patients with renal cell carcinoma, the NLR correlates positively with the level of C‐reactive protein (CRP,^98^ a marker of systemic inflammation that is usually also increased in these patients^99^, ^100^) and is much easier to determine compared with CRP testing. A 1% increase in the NLR corresponds to a 15% increase in the risk of cancer recurrence.^101^ Moreover, an NLR of >1.98 is correlated to a larger tumor size, higher nuclear grade, histologic tumor necrosis, and sarcomatoid differentiation^102^ and associated with pathologic renal sinus fat invasion.^103^ Patients with an NLR of >4.0 have a worse prognosis.^102^ Neutrophils as a predictive factor for cancer is not only that NLR can predict the outcome of cancer, but also that absolute number can be predictive to the effect of treatment. Bevacizumab (Bv), which targets on vascular endothelial growth factor (VEGF), a factor that recruits neutrophils expressing proangiogenic matrix metallopeptidase 9, is frequently used for glioblastoma (GBM) patients. The prognostic value for these patients can be basal neutrophils and Tregs. Counts below 3.9 giga per liter (G/L) for neutrophils and above 0.11G/L for Tregs showed prolonged survival. Neutrophils count is highly associated with response to Bv only in steroid‐free patients, whereas Tregs count is irrelevant to steroid.^104^ NLR is directly associated with the clinical outcome of patients with cancer, which is usually combined with lymphocyte‐to‐monocyte ratio for evaluation.^105^ Sometimes monocyte–neutrophils ratio is also utilized,^106^ whereas it is important to have an absolute NLR value for patients to evaluate cancer stage, adequate treatment, and estimate clinical outcome, which would be largely valuable information. Besides, it seems that absolute number of neutrophils might be an underline factor to select the best treatment, which might be a promising index in clinic.

It is also significant to explore the potential targets on neutrophils to hamper cancer cells proliferation and metastasis. Neutrophils or neutrophils‐related targets CXCR2 inhibitors or anti‐Ly6G together with checkpoint inhibitors have been suggested to use in clinic.^58^, ^107^

## 6 CHALLENGES

As one of the intratumoral immune cells, the importance of neutrophils in cancer development has been revealed by many recent studies, where they have been found to support tumor growth, enhance tumor cell proliferation, and escort CTCs for metastasis. However, the neutrophils have also shown suppression function in the early stage of tumor development. While considering the dual role of neutrophils in cancer development, it is a challenge to find an ideal target on neutrophils. Advanced genomic and epigenomic single‐cell sequencing analysis together with multiple‐photon imaging and mass cytometry may discover new subtypes and novel functions of neutrophils. Manipulation of neutrophils through genetic approaches including CRISP/Cas9 or other gene‐editing techniques to promote their antitumor ability while inhibit their protumor activity targeting therapeutic potential needs further investigation.

## AUTHORSHIP

W.L. and J.C. contributed equally to this work and share the co‐first authorship. H.D. and X.X. share the co‐corresponding authors.

## DISCLOSURES

The authors declare no conflicts of interest.
